# Prevention of Dental Caries

**Published:** 1914-06-15

**Authors:** F. Winchester


### PREVENTION OF DENTAL CARIES.

BY F. WINCHESTER, D.D.S.

Read before the Jackson, Mich., Dental Society, April 1, 1914.

This is a big subject to treat adequately in a short paper, and it is quite presumptious for one to undertake who has only worked along the practical every day lines, therefore, whatever I present will be from practical observation and the results of other men's work.

The theme of this paper ought to be the slogan of every dental practitioner and its title should be framed and hung on the walls of every dental office in the country.

I am not sure but that such a placard placed in public places would produce as much good to humanity as the "Swat The Fly" placards do.

It is claimed and we all believe that dental caries is the most prevalent disease that mankind is subject too, and we sometimes presume to make the statement that it is a preventible disease, but how to prevent it is the problem that we have not as yet solved.

With more than 90 percent of the school children affected and many suffering with decayed teeth, and the number of adults as scarce as hen's teeth, who haven't at present some decayed teeth or filled ones in their mouths, it does not look as though we were making much progress along the line of prevention.

The layman might be justified in telling us that our talk about Prevention of dental caries was pure theory, or to use a pet street phrase, hot air.

The history of dental caries extends back to the ancients. Dr. Pickerill in his work, Prevention of Dental Caries and Oral Sepsis states that 1500 years before the Christian Era the Egyptians used preparations to strengthen their teeth and prevent decay, and at that time keeping the teeth clean was recognized as a preventive measure for tooth decay. So the theory of prevention and the decay itse lfis not of recent origin.

Dr. Pickerill gives a long list of data and methods from the above mentioned time down to the present, and practically all of them recognized the fact that to preserve the teeth they must be kept clean.

In the fifteenth century a code of rules was published by Pierre Fauchard for the care of the teeth. The teeth are to be cleaned with a stick after each meal; the mouth to be washed out with an aromatic decoction; a dentifrice should be used the last thing at night. Compare the above with the nineteenth and twentieth century teaching, what difference do you discover? Scarcely any except perhaps in details.

It would seem that 400 years ago there were just as good theories advocated, and no doubt practiced, as today. Prophylaxis was then the watchword and it has remained so, except perhaps that today we emphasize its importance.

Much, however has been learned along the line of the cause of caries, and very much remains to be learned before we may hope to prevent dental caries to any appreciable extent. The profession is not in a position to educate the public until it first educates itself as to a practicable means of prevention.

At present our work is along the line of prevention, either directly or indirectly.

Every tooth rescue or restoration we make ought to be with the end in view of making the mouth more sanitary and thereby preventing further decay.

The time has arrived when the dentist must be something more than a tooth tinker. Too often we make the mistake of treating individual teeth, instead of the mouth as a whole.

There is no particular benefit to be derived by caring for one or more teeth just because they show, and leaving others that are out of sight with cavities filled with enough bacteria to corrupt the whole oral cavity. We have not done our full duty to our patients if we discharge them with the idea that their mouths are in proper condition when there are old fillings with bad margins, crowns that do not fit at the cervix, teeth that need extraction, or until every tooth is in a condition that it may be properly cleaned and the mouth kept sanitary.

Decay and filth go hand in hand and until the dentist awakes to the possibilities of clean mouths and solves the problem of keeping the teeth clean and intact, prevention of caries will not be accomplished.

Dr. Black has given us a system of cavity preparation, that if carried out will do much to prevent recurrent decay and oral sepsis. We have no excuse at the present time for having fillings that overhang the cervical border, or for ignoring the interdental space and its protection.

Some one may say that so few persons appreciate our efforts that it is not worth while to do our best, but my opinion is that when we start down *The Don't Care Road* we gain momentum so rapidly that sooner or later we find ourselves caring for nothing but the dollars that we receive as fees.

In the prevention of dental caries there are two conditions to attain that are fundamental.

First: With the consent and aid of the patient we should put the teeth and mouth in as nearly a perfect condition as possible, and, Secondly, teach and assist the patient to keep the teeh clean and the mouth healthy.

Not every patient will consent to treatment extensive enough to accomplish the first, and but few will appreciate what we are endeavoring to accomplish enough to follow out our instruction when honestly given, and here is where we fail if we are not wise enough to give instruction and advice that will produce results.

We have all accepted the theory that decay is caused by the action of lactic acid. That is starches (sugar, etc.), adhere to the teeth forming mucine plaques that act as culture media for bacteria, which under favorable conditions are produced in sufficient quantity to produce an acid that acting on the enamel dissolves it until the acid reaches the softer structure, the dentine, forming larger and ever increasing culture beds and the final breaking down of the entire tooth crown structure.

We have also accepted the theory that to combat this acid we must use alkali mouth washes and dentifrice of alkali reaction and some so-called antiseptic, thrown in to insure results, but recently Dr. H. P. Pickerill of New Zealand, and Dr. W. J. Gies of the Department of Byological Chemistry of the Columbia University, two men on opposite sides of the globe, working for years independent of each other, have both arrived at the conclusion that dentifrice and mouth washes of alkaline reaction produce directly opposite results from what we expect and that fruit acids are the logical dentifrice.

Research work has proven to them that fruit acids dissolve or break up the mucine plaques and the fruit of proper strength acid becomes an active salivary stimulant producing such a flow that the debris is washed off or flushed out of the cavity. And also further that after a few moments the saliva becomes abnormally alkali, neutralizing the fruit acid in the mouth, thereby protecting the teeth from harm by caries.

While on the other hand alkali solution and chalk, etc., produces a more sticky mucine and also retards the flow of saliva. Dr. Pickerill in his work gives tables of comparison of normal saliva and the results of increased alkalinity after using certain fruit acids and both conditions are very masked, especially after using oranges or apples, showing increased flow and markedly increased alkalinity.

Dr. Gies suggests and advises the use of diluted cider vinegar—one part vinegar and two parts water—these to be used when brushing the teeth.

Dr. Pickerill suggests acid potassium tartrate (cream tartar), this he claims is an active salivary stimulant. He says he is convinced that no harm can come to the teeth from the acids when used in such a strength that they are active salivary stimulants.

Potassium tartrate is soluable in only one to two hundred of water, so there is no danger in using it in the liquid form.

These suggestions sound rather irrational after being educated to the use of alkaline dentifrice, and our belief that all acids effects the teeth. It seems strange, however, that two men so remote from each other and working independently of each other should arrive at this same radical conclusion.

We must admit that under our present alkaline theories dental caries is not decreasing very rapidly, and if these men perchance may be on the right road, we at least ought to bid them God speed.

Surely much credit is due men who are doing research work, and the dental profession has need of much work of this kind.

We can do no less than at least accept their theories and act on their suggestions, and endeavor to prove out in actual ractice the results of their findings.

Certainly a great amount of research work must be done by the few before we can prove the assertion that dental caries is a preventable disease and bring the method of prevention within the scope of the masses.

DISCUSSION.

Dr. H. F. Parks: I have often seen real unhygienic conditions of the patients mouth with little or no caries. I once carefully cleaned a patients teeth so thoroughly that they became very sensitive, and the patient complained for months that I had done him an injury rather than helped to prevent caries. I remember another case where the patient had never brushed his teeth and there was no indication of caries anywhere. Isn't it possible that in some mouths what seems to be a menace to the teeth is really a protection against caries? I have never been able to determine by a microscopic inspection of the saliva that certain conditions of saliva predisposed the teeth to caries. I have never been able to determine that there was more decay in the mouths containing the thick and stringy saliva than where the saliva was thin and watery. I wish we could know some of these symptoms more definitely, because I find it embarrassing not to find the conditions we are taught to expect from these signs.

Dr. A. B. Green: I do not think because we find an occasional mouth that is filthy and yet has no caries that we are justified in neglecting surgical cleaning of the teeth, because patients can not keep the surfaces of their teeth as free from extraneous deposits of a temporary character, and especially when the teeth are covered with calcular deposits, or are in irregular positions, or are filled carelessly. Then, too, I believe the very act of brushing the teeth and gums by the patient at stated intervals is helpful in increasing nutrition. No mouth washes or medicated applications are so valuable as regular and effective brushing with the tooth brush and a suitable dentifrice.

Dr. C. J. Lyons: The prevention of dental caries is almost too big a subject for us to know it in all its many varieties. We do a good deal of talking about it, and most of out efforts are made to restore its ravages and to give that part of the tooth that is left a chance to keep up normal function. We can do much by repairing the teeth so thoroughly that the patient can use them so effectively that his general health will be so good that the saliva and mouth juices will be normal and not a menace or contributing factor of caries. We should not only do the surgical and prophylactic work thoroughly but we must instruct our patients how to do their part and make sure that they are faithful in doing what we tell them—they must do it if they expect to escape dental caries. I am certain that not only theoretically but practically it is possible to eradicate all caries and to keep the mouth practically aseptic.

Dr. N. S. Hoff: Discussed the old theories of caries from the Chinese and early Greeks to the Miller microorganisms. He explained the manner in which the micro-organisms secreted themselves under the protecting environment of the insoluable plaques, where by their functioning they caused fermentative products which destroyed the calcific material of the tooth; always secreting themselves in the crown fissures or the obscure interdental spaces where mastication or prophylaxis could not disturb them. It is this tendency for the collection of the micro-organisms in the obscure places that make the demand for professional prophylaxis, as the ordinary dental toilet by the patient is not radical enough to break up these nests of caries producing influences. By periodic surgical cleansing of the tooth surfaces the patient is able to keep the teeth entirely free from deposits that will harbor and encourage fermentation and the action of resultant acids which produce the initial lesions of the enamel. We all know how rapidly a tooth will be destroyed once the enamel is perforated, and we all know that then the only prophylaxis is in filling the cavity and stopping the progress by an insoluable restoration of the tooth surface. If we could obliterate the deep fissures in tooth crowns and protect the proximal surfaces from collecting deposits we should have very little tooth caries, as the tongue keeps the lingual surfaces fairly clean, and the lips and tooth brush keep the labial and buccal surfaces free from deposits.

Dr. F. W. Howlett: There is so strong a tendency for the teeth to decay in some mouths that no surgical or prophylactic measures will successfully cope with it. Some one has said there are seven ages of teeth as of man, and during the age of youth and childhood the teeth probably suffer from caries more than at any other time, whether this is because the teeth are less resistant at this time or their environment is more unfavorable is an unsettled question. There are also periods of especial stress when the teeth seem to suffer more than usual. I sometimes think it impossible to educate all patients to the point where they will do their part in prevention. I believe that certain forms of over stimulation of the flow of saliva produces vitiated secretions which collect on the teeth and supply the culture medium for the acid fermentation. We have hoped that our scientific workers would find a cause for caries in the saliva, but so far nothing convincing or even promising has been developed. We find caries so frequently associated with bad health that we are almost forced to believe that the disease has a systemic cause or relation at least. Just what this may be no one has as yet given us a reasonable clue. The work of Pickerill is a most interesting and valuable contribution to our knowledge of this important question, but it does not demonstrate a satisfactory theory as to the cause of caries or give us an idea for its prevention. The truth is the matter is so complex and so intimately associated with our normal and pathological functions that it will probably be a long time before we reach any decided conclusion as to its cause and treatment.

Dr. W. E. Merrit: I have noticed many times that there was less caries in the mouths of patients whose saliva was thin or watery in character. I have thought that such saliva did not furnish so much material of a fermentable character to lodge about the teeth and so supply the means for producing acids to attack the teeth. Possibly it may have a more antiseptic or disinfectant value than the thick and ropy salivas. I am a strong advocate of vigorous brushing, not only of the teeth, but of the gums as well. I believe we must begin early with children and get them to form correct habits of brushing their own teeth, and of coming for professional treatment at more frequent intervals, that we may practice more preventive and less rescue dentistry.

Dr. C. B. Blackmarr: If we are to prevent dental caries we must necessarily look farther than the tooth itself. The environment in which the tooth is found is most favorable to its destruction. All the necessary conditons for fermentation are constantly present in the mouth. We have the infective micro-organisms and the moisture and temperature with the pabulum on which it thrives, all in abundance and under most favorable conditions. Then the saliva is a normal secretion of the salivary glands and while at times it may have a protective influence, at others it may be a contributing factor. So that our problem is necessarily a most complex one, and will require our most profound study to make it so simple as to be easily comprehended or to be successfully treated. At present it seems the best thing we can do is to keep our patient informed as to the best methods for them to use in prevention and we must do our best to restore the damages. Bad health has a large influence no doubt, and so we must advocate good health. Sanitation of every kind as well as oral sanitary measures are necessary.

Dr. E. B. Hosem: As the essayist has said, it is all well enough for us to talk about educating the public, but if we ourselves don't know, how shall we educate the people. Multiplication of mouth toilet articles, such as brushes, dentifrices, mouth washes, etc., have done some good, but these things we all know are inadequate. We have tried oral prophylaxis and it has done much to justify itself, but when we stop to think how few of all the people can have this treatment adequately, if at all, we see where it fails to give the necessary remedy. For the present it seems that we shall have to supply the best treatment we know of and keep up the propaganda of educating the people, and pray that the Lord will send us more dentists, or that our scientists will discover a means of controlling this most disastrous disease.

Dr. J. R. Foreman, of Clinton: I want to thank this society for the privilege I have enjoyed this evening, it has been a great pleasure to hear the paper and discussion of this important subject, and to hear so general and satisfactory a discussion. I don't believe I ever attended a dental meeting before, out of which I have gotten so much as tonight. I practice in a small town as you know, and I have my troubles in the educational line, I assure you. It may be difficult as you say to carry out a rigid system of prophylaxis in Jackson, but I can assure you that you don't know what trouble is. Why, if I were to spend a half day treating a patient for pyorrhea, or in cleaning teeth, and charged five dollars for it, I would have to leave town. Why last week a farmer brought his little boy to me and I extracted two deciduous teeth for him, and charged fifty cents for the service. The father became very angry and called me a robber, and said he would establish a boycott on my office in his neighborhood. Now what chance have I to make any progress in preventive dentistry with such people?

Dr. Winchester: In closing this discussion I must thank those who have so kindly and amply discussed the subject and my paper. As I said in my paper the subject is so large and important that we are all interested, and we all have some ideas about it. This fact is the hopeful sign that we shall know more about it, and I hope we shall all keep working and thinking, and also that we shall do what we can to encourage our scientific research men who are to bring us to the knowledge necessary to solve the problem.

